# Engineering a Biocompatible Scaffold with Either Micrometre or Nanometre Scale Surface Topography for Promoting Protein Adsorption and Cellular Response

**DOI:** 10.1155/2013/782549

**Published:** 2013-02-27

**Authors:** Xuan Le, Gérrard Eddy Jai Poinern, Nurshahidah Ali, Cassandra M. Berry, Derek Fawcett

**Affiliations:** ^1^Murdoch Applied Nanotechnology Research Group, Department of Physics, Energy Studies and Nanotechnology, School of Engineering and Energy, Murdoch University, Murdoch, WA 6150, Australia; ^2^Division of Health Sciences, School of Veterinary and Biomedical Sciences, Murdoch University, Murdoch, WA 6150, Australia

## Abstract

Surface topographical features on biomaterials, both at the submicrometre and nanometre scales, are known to influence the physicochemical interactions between biological processes involving proteins and cells. The nanometre-structured surface features tend to resemble the extracellular matrix, the natural environment in which cells live, communicate, and work together. It is believed that by engineering a well-defined nanometre scale surface topography, it should be possible to induce appropriate surface signals that can be used to manipulate cell function in a similar manner to the extracellular matrix. Therefore, there is a need to investigate, understand, and ultimately have the ability to produce tailor-made nanometre scale surface topographies with suitable surface chemistry to promote favourable biological interactions similar to those of the extracellular matrix. Recent advances in nanoscience and nanotechnology have produced many new nanomaterials and numerous manufacturing techniques that have the potential to significantly improve several fields such as biological sensing, cell culture technology, surgical implants, and medical devices. For these fields to progress, there is a definite need to develop a detailed understanding of the interaction between biological systems and fabricated surface structures at both the micrometre and nanometre scales.

## 1. Introduction

The last two decades have seen a tremendous level of fundamental research and development into nanotechnology. Recent developments in material science, engineering, biotechnology, and biomedical fields have clearly demonstrated the many potential applications of nanotechnology [1, 2]. The basis of this intense nanotechnology-based research is derived from the fact that nanoscale matter can have significantly different properties than its bulk counterpart [3, 4]. The discovery and investigation of these unknown properties, using new advanced characterization techniques, have the potential to deliver detailed information that can be used to develop many new nanotechnology-based applications. These new characterization techniques have come about from the development of the atomic force microscope (AFM) and the scanning tunnelling microscope (STM) in the 1980s [5]. Both these techniques have given researchers the unprecedented ability to explore and chart the properties of these newly created nanomaterials. These newly discovered nanomaterials have the potential to revolutionize many current pharmaceutical and biomedical applications; and along the way they have the potential to generate new superior tools to assist in current therapies and provide the foundations for new avenues of biomedical intervention in the near future.

Currently, there are a number of processing techniques capable of producing nanomaterials, but recent studies have focused on refining these processes to produce new nanoscale materials. A few processes that are currently being investigated and refined to produce high-quality nanomaterials are chemical vapour deposition to produce carbon nanotubes and carbon nanostructures [6, 7], ultrasound techniques to produce nanohydroxyapatite crystals for biomedical applications [8, 9] and the wet sol-gel synthesis method for creating iron oxide (Fe_2_O_3_) nanoparticles [10, 11]. The most attractive feature of using nanotechnology-based processing techniques is that it gives the manufacturer far greater control over the polydiversity, phase, crystalline structure, topography, morphology, and quality of the nanomaterials produced.

**Figure 1:**
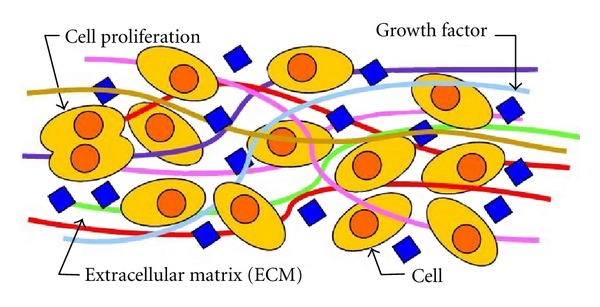
Schematic of the cellular environment.

From a biomedical point of view, the cell is the basic unit of a biological system and every organism either consists of cells or is itself a single cell [12]. While cells are generally in the micrometer-size range, their component structures and associated environment are generally in the nanometre to submicrometre range. In fact, the molecular building blocks of life, such as proteins, carbohydrates, nucleic acids, and lipids, are all nanometre scale structures. And from the cellular perspective, the interaction between the cell and nanometre scale structures such as proteins are crucial for controlling a variety of cell functions such as proliferation, migration, and the production of the extracellular matrix (ECM) (Figure 1) [13]. In addition, the physical structure and chemistry of the nanometre scale structure directly influence the behaviour of the cell in contact with the surface of the nanometre scale structure. For example, when a biomaterial comes into contact with the internal cellular environment of the body, proteins spontaneously adsorb onto the surface. This results in the formation of a surface-bound protein layer, which mediates between the biomaterial surface and the cell surface receptors during subsequent cell attachment. How the geometrical and chemical properties of a biomaterial surface influence the adhesive attachment of the cell to the surface and its subsequent influence on the proliferation of anchorage-dependent cells is still an area of active investigation. Furthermore, the adsorption of proteins to the surface of nanometre scale structures is highly dependent on the nature of the surface; for example, surface charge, surface chemistry [14], wettability [15], surface density of cell-binding ligands [16], and nanotopography [17] all play an important role in determining the cell-substrate interaction. In particular, cells are highly sensitive to the local nanotopographic landscape of the ECM and the substrate. For example, Yao et al. have shown that the nanometre topography of bioimplant materials such as Titanium (Ti) and its alloys (Ti_6_Al_4_V and anodized Ti) can enhance the adhesion between osteoblast cells and the surface of the underlying substrate [18, 19]. Similar studies by Webster et al. have also revealed an enhanced attachment function between osteoblast cells and nanoceramic surfaces [20].

## 2. Tissue Engineering

The field of tissue engineering came into existence during the mid-1980s to address the high demands for regenerated tissues in clinical applications. Its creation resulted from the convergence of several scientific, technological fields, biotechnology, and medicine. Tissue engineering is still an evolving field whose primary function is to recreate the appropriate signals to cells that promote biological processes, which can then create new and/or repair damaged tissues by rational design. According to Langer and Vacanti, tissue engineering is a highly interdisciplinary field that combines engineering principles, biological sciences, and medicine toward the development of biological substitutes to restore, replace, maintain, or enhance tissue and organ function [21, 22].

Development over the past few decades in this field has produced engineered implantable human tissues such as bone, cartilage, and skin [23, 24]. Currently, there are clinical trials underway that are investigating the feasibility of using tissue engineering techniques to produce a human bladder and blood vessels [23]. The research to date has clearly demonstrated that a major function of tissue engineering is to create an environment that can promote productive and efficient cellular activity; however, this environment is influenced by a number of tissue-dependent factors. A recent study by Yang et al. has revealed that tissue engineering is composed of four key factors: (1) cells, (2) scaffolds, (3) bioreactors, and (4) signals [25]. To obtain the most beneficial and effective outcome, an exhaustive examination of these key factors is needed to achieve the most appropriate contribution from each step for the particular tissue being addressed. This equates to determining, developing, and instituting the most promising environment that can support optimal survival conditions for the tissue undergoing regeneration. The first step in any tissue engineering process involves the harvesting of appropriate cells from donor sites and then introducing these seed cells into a suitable scaffold structure contained within a suitable growing medium. The biocompatible scaffold structure provides a 3-dimensional (3D) environment which promotes cell attachment and proliferation [26]. Apart from being 3D, the scaffold should be made from a biocompatible degradable nontoxic material and should be highly porous to permit the diffusion of nutrients, oxygen, and waste products [27]. This is where nanotechnology can have a significant role to play, since nanotechnology permits the creation of a specialized scaffold structure that can be specifically designed for the particular cell or tissue type. Furthermore, the scaffold can be enhanced to provide the maximum environmental conditions for optimal cellular growth. 

Many studies have shown that cells in general tend to behave more naturally when they are cultivated in a 3D scaffold environment [28]. This has resulted in the design of 3D scaffolds that incorporate tissue-specific topographical and environmental enhancements. These enhancements are capable of creating an appropriate microenvironment that can support, regulate, and assist cell function. For example, cells that were cultivated on scaffolds containing 10–100-*μ*m-sized ridges and grooves promoted elongated cell growth that was orientated in the direction of the surface feature [29]. Cells in their natural tissue environment are generally surrounded by the ECM, a structure composed of many interwoven fibrous molecules, which forms the architectural structure capable of supporting and directing cell behaviour via cell-ECM interactions. The natural scaffold structure of the ECM is a complex formation of architectural features such as fibres, pores, and ridges that vary in size, which are capable of providing physical signs that can directly affect cell behaviour [30–33]. The ECM is composed of proteins such as fibrous collagen, fibronectin, proteoglycans, laminin fibres, and hyaluronic acid, which gives the natural scaffold its chemical, mechanical, and topographical signals that are needed to influence cell behaviour [34]. Therefore, it is important when developing 3D scaffolds that the scaffold biomimics features of biological tissues either compositionally or structurally so that the scaffold can replicate the regeneration process in a similar way to nature. For example, the scaffold topography can directly influence growth parameters responsible for cell adhesion, apoptosis, differentiation, genetic expression, migration, morphology, orientation, and proliferation [35, 36]. 

In particular, topological features provide contact guidance for the cell, which influences the cytoskeletal arrangement and adhesion of the cell [37]. Historically, Harrison was the first to observe the interaction between a substrate's topography and cellular tissue when investigating spider silk fibres in 1911 [38]. The nerve cells tended to grow in a bipolar shape orientated along the fibres. The term “contact guidance” was later coined by Weiss in 1945 to describe the effect of the fibres on cell orientation and proliferation [39, 40]. Recently, Allmeling et al. have investigated the column growth of Schwann cells along the length of spider silk fibres for a possible nerve conduit and found that the structure promoted successful cell adhesion and migration [41]. Studies of other cultivated cells on different planar scaffold materials, containing arrays of micrometer-sized protrusions have promoted cell attachment and reduced cell proliferation [42]. These studies have clearly demonstrated that controlling the surface features of the scaffold in a specific way can directly influence cellular adhesion, protein absorption, proliferation, and morphology. In addition, cells also have the ability to transform their microenvironments on the scaffold structure by changing the ECM it produces. This can be done by synthesizing or degrading the ECM, secreting cytokines, and communicating with other cells and matrix on the scaffold by molecular and physical signals [43]. It is clear that the interaction between the individual cells, the ECM, and the nanotopographic surface features of the scaffold is a dynamic process and is crucial to fully understand the cellular response as a whole in developing suitable biomaterials for tissue engineering.

## 3. Construction of a Tissue Scaffold

The scaffold architecture used in tissue engineering has three distinct size ranges. The first is the superstructure; this size range essentially covers the overall shape and dimensions of the scaffold. The second is the microstructure, which refers to the scaffold surface features at the cellular level and final size range is the nanostructure, which refers to the subcellular features of the scaffold surface [35]. Generally, biomaterials used in tissue engineering applications such as tissue repair and regeneration have generally favoured bioinert materials for permanent bioimplants such as hip and knee replacements [44]. In the case of scaffolding materials, both natural and inorganic, including metal oxides, have been investigated [45]. The selection of the material used to manufacture the scaffold is an important factor in its successful application. A wide variety of natural biodegradable materials have been extensively studied for potential use in tissue engineering since the body's natural pathways can effectively deal with the by-products resulting from their breakdown. Natural polymers such as polysaccharides [46–50], chitosan [51–56], hyaluronic-based derivatives [57–60], and protein-based materials such as fibrin gel [61, 62] and collagen [63–66] have all shown some positive outcomes during investigative trials. However, on the whole, these polymeric materials lack sufficient mechanical strength to effectively support tissue growth in the body environment. On the other hand, inorganic or synthetic biodegradable polymers have been fabricated under controlled conditions to produce a variety of scaffold structures with selectable, predictable mechanical, and physical properties.

Biopolymer materials are composed of simple, high-purity constituent monomers. An advantage of biopolymers is that they have a controllable degradation rate within the body's environment and their reactions to the body fluids during degradation produce low toxicity by-products that can easily be handled by the bodily excretory functions. Examples of these bulk biodegradable polymers include poly-(lactic acid) (PLA) [67–72], poly(l-lactic acid) (PLLA) [65, 73–75] poly(lactic-*co*-glycolic acid) (PLGA) [76–79], polycaprolactone (PCL) [74, 80–82], and poly(glycolic acid) (PGA) [83–86]. These are generally poly-*α*-hydroxy esters that de-esterify in the body environment as the polymer slowly degrades to produce simple metabolites [87]. An example of biopolymers currently in use is the biodegradable sutures composed of PLA and PLG that are employed in medical procedures. Because polymers are such good biocompatible materials, they have also been extensively investigated for the controlled delivery of drugs to specific organs within the body [88–90]. In general, polymers are strong and can be fabricated into a variety of different shapes and structures, such as, disks, fibres, films, and pellets, as required for the specific application. In addition, they can be produced with microtypographical surface features that can effectively induce physical cues that can enhance cell interaction with the surface of the scaffold.

Another promising material with new and novel properties for biomedical applications is polyhedral oligomeric silsesquioxane (POSS). The material structure consists of an inner inorganic framework of silicon and oxygen atoms which form a nanometre size cage. Surrounding the cage is an outer shell of organic groups which can be composed of hydrogen, alkyl, alkene, and arylene. The biomedical applications of POSS arise from the materials biocompatibility, biostability nontoxicity, cytocompatibility, and resistance to degradation [91–93]. It is due to these properties that POSS has been incorporated into wide range of nanostructured copolymers for a number of biomedical applications such as biomedical devices (heart valves, coronary stents) [94, 95], drug delivery [96], and tissue engineering [92]. 

Inorganic materials such as bioglass, ceramics, and metal oxides have also been investigated for possible use in tissue engineering applications. This research stream stems from the fact that despite the successful application of polymers, there are still some unresolved issues that need to be resolved. The first issue stems from the local inflammatory response of the surrounding tissues to the presence of the polymer material, and the second results from the uneven degradation process of polymer used in the scaffold. In spite of this, polymer scaffolds are superior to both ceramic and metal oxides for soft tissue applications such as skeletal muscle, cardiovascular tissue, and skin substitutes. The advantage of using polymers in soft tissue applications stems from their close chemical and physical similarity to natural cellular tissues [97–99]. Studies using bioactive glass as a scaffold material have revealed that when the glass was seeded with osteoblasts, there was an enhancement of cellular proliferation [100]. Furthermore, metals such as pure tantalum (Ta) have also been successfully used to produce tissue scaffolds for the adhesion, growth, and differentiation of osteoblasts. One innovative technique used to create a Ta scaffold begins with the pyrolysis of polyurethane foam [101]. The foam turns into a low-density carbonaceous skeleton composed of a repeating dodecahedron structure that produces an interconnecting array of pores. In the next stage, a chemical vapour deposition/infiltration technique (CVD/CVI) is used to deposit pure Ta onto the carbon skeleton and produce a porous metal scaffold. The structural integrity of the scaffold increases as the deposition process continues. An advantage of this deposition process results in the formation of a crystallographic growth pattern that orientates the Ta layer to form a microtextured surface, which is similar to cancellous bone [102, 103]. In addition, by changing the characteristics of the precursor polymer used and the thickness of the Ta layer deposited onto the carbon skeleton it is possible to control the size of the pores produced. For orthopaedic applications, the thickness of the Ta layer ranges from 40 to 60 *μ*m, while the pore size ranges from 400 to 600 *μ*m, and the resulting scaffold porosity can vary from 75% to 80%. The high porosity and large pore size are ideal for deep and extensive vascular tissue penetration, which results in strong tissue attachment strengths [104]. This type of vascular tissue penetration and subsequent growth has also been seen in images of highly porous alumina ceramic foam metals [105]. Furthermore, studies into the growth of osteoblasts on metal oxide surfaces such as nano-porous alumina have also shown a positive response [106, 107].

### 3.1. Important Parameters Needed for a Successful Scaffold Structure

The operational demands that are placed on a substrate's scaffold structure when implanted into the body environment are numerous, and the scaffold must overcome many challenges to achieve a successful clinical outcome. For example, the biocompatibility of the scaffold material is crucial in preventing any cytotoxicity, immunological reactions, and inflammation responses from the body [108–110]. This is particularly important since the presence of any scaffold material within the body environment will initiate an inflammatory response at the scaffold site. As a consequence, a complex biochemical cascade of events takes place in which cells arrive and start producing chemokines, cytokines, and growth factors to initiate the repair of damaged tissue surrounding the scaffold site. The presence of these cells on the surface of the scaffold can initiate a foreign body reaction to biomaterial used to manufacture the scaffold. These cells produce oxygen radicals and enzymes that have the potential to degrade the scaffold which can ultimately lead to the failure of the scaffold [111]. Recent *in vitro* and *in vivo* studies by Lamers et al. have revealed that the immunological response to a biomaterial surface could be altered by introducing nanometre sized grooves to a substrate surface [112]. The nanometre patterned surfaces were found to solicit a response from murine macrophages (cell line RAW264.7) which resulted in an altered gene expression and protein secretion after 24 h *in vitro*. However, there was no noticeable change in protein secretion during the *in vivo *study. These studies highlight the importance of using a biomaterial with appropriate surface topography to solicit a favourable immunological outcome, since the macrophages were clearly sensitive to the nanometre scale groove features and may assist in the healing process. The scaffold provides the initial framework for the seeded cells to attach, proliferate, and differentiate. During this process, the initial scaffold mimics the ECM environment, and as new ECM, is being created by the cells, it will provide integrity to the new tissues as the scaffold slowly degrades over time. The surface chemistry of the scaffold material is an important factor during the formation of new ECM, since the scaffold must be chemically compatible with the ECM. Since the ECM is nature's own tissue scaffold and forms the cell environment, it is desirable that any engineered scaffold biomimics the ECM as close as possible. This is because the chemistry and topography of the ECM provides the cues that initiate and modulate cell adhesion, cellular interaction, proliferation, and migration [30, 64, 113–118].

In exploring their surrounding environment, cells receive a variety of complex biochemical and biophysical signals via filopodia at the cell boundaries which spread out over the surface of the ECM [117]. To accomplish a number of biological processes, cell movement, and migration must take place. The cell achieves motion through the action of protrusions from the cell membrane, forming integrin adhesions combined with cellular contractions. The direction of cell motion is guided by environmental stimuli such as biochemical and biophysical signals. In addition, cells also respond to mechanical stimuli in the form of gradients in the mechanical stiffness or rigidity of the substrate—a phenomena known as durotaxis that results in cell membrane stretch, compression, and interaction with the surface topography [119–121]. Cells that come in contact with stiff substrates develop strong focal adhesions that securely anchor the cell to the substrate. This is in contrast to soft substrates which induce small, transitory adhesions that are unstable and provide weak anchorage for the cell. In effect, durotaxis creates a bias that influences the direction of cell migration from softer substrate regions towards regions that are characterised by increasing elasticity or stiffness [122]. For instance, multipotent mesenchymal stem cells (MSCs) display lineage-specific differentiation when cultured on substrates that mimic the stiffness of native tissue environments. In the case of MSCs cultured on substrate that mimics the bone environment, the cells become osteogenic. While MSCs exposed to substrates that mimic a myogenic tissue environment become muscle cells [123, 124]. 

In addition, the biomaterial needs to be able to prevent any rapid bulk degradation effects that might result in the formation of voids and defects within the scaffold structure. Another important property of a biomaterial is its ability to be easily sterilized prior to its application without any significant changes to its surface chemistry [64, 125–128]. Also, the biomaterials used in the construction of the scaffold should not be hydrophobic, since the wettability of the material is an important factor that must be carefully considered for successful cellular adhesion and attachment. At the molecular level, it is extremely important that the scaffold contains a network of pores and interconnecting channels to facilitate the diffusion of nutrients, oxygen, metabolites, and waste products. Kim and Coulombe were able to demonstrate that pore size and a high surface area to volume ratio were important parameters that encouraged cell penetration and subsequent growth in the scaffold structure to form cellular associations [129]. In addition, if the scaffold structure is going to be rather large, then the structure should be designed to contain a life-supporting capillary-like network as an integral part of the scaffold. Furthermore, the interactions between the cell micrometre scale topography and nanometre scale topography of the scaffold structure can influence cell attachment and adhesion, proliferation, and migration. A recent investigation by Andersson et al. revealed a link between epithelial cell attachments to surfaces of similar chemistry and that the cell morphology and cytokine production were strongly dependent on the underlying nanometre scale TiO_2_ deposited surface topography [130].

### 3.2. Scaffold Manufacturing Techniques

The cells in the human body are micrometre sized objects, with the largest cells being the anterior horn in the spinal cord (~135 *μ*m) and the smallest being the granule cells in the cerebellum (~4 *μ*m), with a typical cell size between 10 to 20 *μ*m [131]. While the topography created by the ECM proteins are generally in the submicrometre to nanometre size range. This size range has been shown to have an influence over cell behaviour at the cellular level [132]. Since cells are responsive to the topography of the ECM, engineered scaffolds and substrates must also display similar surface topographical features that can provide effective signals to influence cellular behaviour. In addition, the size, density, and distribution of the topographical features found on the surface of the ECM are also dependent upon the types of cellular tissues that form the ECM [32, 133]. Therefore, it is important that the information derived from the specific cell generated ECM topography is translated into an effective fabrication process that replicates these surface features onto tissue culture substrates and scaffolds [134]. 

There are three main categories used to define the type of topographical features that can be fabricated, the first is ordered, which involves the production of accurate repeatable surface features and the second involves the formation of irregular or unordered features such as surface roughening and fibrils [35, 135]. Both of these categories attempt to replicate the topographical features of the ordered ECM, the third involves the removal of cells from body tissues and then use the native decellularized tissues as cell culture scaffolds [136]. 

Biological scaffolds composed of decellularized tissues and organs are capable of providing a natural ECM structure that can be used for a variety of tissue engineering applications [137–139]. The ECM harvested from a number of tissue sources are then used in a number of tissue engineering applications such as blood vessels [140, 141], heart valves [142, 143], urinary bladder [144, 145], small intestinal sub-mucosa [146, 147], liver [148, 149], skin [150], and lung [151]. If properly prepared, the decellularized tissue scaffold can preserve much of the original ECM structure and bioactive functional molecules, which enables the scaffold to provide important physical and chemical signals needed to support cell functions such as attachment, differentiation, and proliferation. The use of biological derived tissues as scaffold structures has the potential to create an effective environment to induce cellular growth. To date, decellularized ECM scaffolds have been described for a number of organs, with an engineered bioartificial heart being investigated *in vivo* using an animal model [152]. 

During the late 1970's and early 1980's with the development of cellular telephones, digital devices and the personnel computer in the electronics industry, it was possible to develop many novel micrometre and later, nanometre scale fabrication techniques to manufacture extremely small and compact equipment. Using these new fabrication techniques in conjunction with the continually developing biomaterials field, many research teams across the world have the potential to fabricate 3D scaffold structures that can effectively biomimic the topographical features of the ECM. Once the specific tissue engineering application has been identified, the required chemical, physical, and mechanical properties can be determined and then a suitable fabrication technique can be selected to manufacture the scaffold structure. 

**Figure 2:**
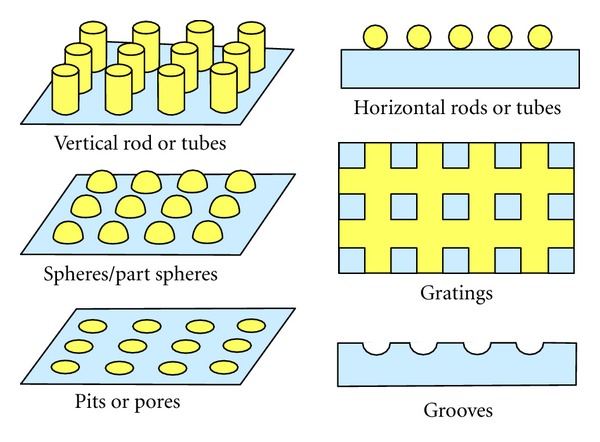
Some typical topographical surface features.

Technologically driven top down micrometre and nanometre scale lithographic fabrication techniques have been used to produce a variety of topographical features such as grooves, gratings, pillars, spheres, pits, and tubes (see Figure 2). These techniques have the ability to control the fabrication of accurate and highly reproducible topographical features over the surface of a variety of substrates. Several lithographic techniques have developed over the years that have enabled researchers to explore a variety of cell-topography interactions on materials such as silicon, polymers, and titanium [132]. Electron beam lithography, which was originally developed for producing integrated circuits has been successfully used to produce both micrometre and nanometre scale topographical features on the surface of several biocompatible materials. However, this technique is relatively expensive and time consuming due to substrate exposure time, which limits the production of substrates and scaffolds [153]. Photolithography is a process which selectively removes predetermined material from the surface of a substrate to fabricate micrometre topography. The first use of this technique for cell response studies was made in the early 1980s when a series of micrometre scale grooves were formed in the surface of a silica substrate [154]. Other techniques such as X-ray lithography, laser ablation, nanoimprinting, and microcontact lithography have also been used effectively used to produce topographical features on a variety of materials.

Two recent techniques that may provide improved routes for the fabrication of 3D cellular scaffolds for a variety of tissue engineering applications are self-folding materials and multiphoton lithography. Micrometre scale fabrication of 3D structures by controlled out of the plane self folding of 2D patterns using a laser direct-write-based technique has the potential to create elaborate and precisely structured substrates that can be used to model *in vivo* cell behaviour [155]. The Japanese art of paper folding known as origami transforms 2D patterns into 3D shapes. A similar approach uses self-folding, a self-assembly process in which planar structures fold up from the 2D substrate when exposed to specific activation stimuli [156]. For example, laser origami uses a laser direct-write technique to produce feature through laser fabrication of the pattern in the substrate, then deposit the active elements on the pattern via laser transfer or cutting and finally the substrate is heated using the laser which results in the cut patterns folding up out of the substrate [156]. The other recent technique is Multiphoton lithography (MPL) which can be used to fabricate 3D tissue scaffold structures. It is a computer controlled fabrication technique in which the morphological features of a biological structure are programmed as stack of 2D tomographic cross sections. The cross sections act as input for a sequence of reflective photomasks that are used to direct the replication of the biological structure. The direct-write process, using a laser, translates the programmed data from the cross sections into a protein-based 3D reproduction of the original biological structure [157]. This technique is capable of producing complex 3D structures with well defined architectural features such as size, shape, inter connectivity, branching, geometry, and orientation that have the potential to mimic complex 3D biological structures [157]. Many of these top down techniques tend to be very expensive since they require specialised instrumentation and equipment, complex ultrahigh vacuum systems, and clean room facilities. 

On the other hand, the electrospinning process, which was originally developed by the textile industry, has been used for the past 100 years. Refinements of the technique in the last decade have seen it increasingly used in the manufacture of fibre with diameters ranging in size from many microns to tens of nanometres [158]. The fibres produced are used to form a nano-fibrous porous structure that permits cells to enter the scaffold, while allowing the flow of nutrients and waste products from the cells [159, 160]. Scaffold structures of synthetic polymers such as PLA and PLGA, and natural polymers such as collagen (types I, II, III), chitosan, elastin, fibrin, and silk have been produced using this technique. Agarwal et al. have demonstrated that polymeric materials manufactured using this technique induce a favourable and conducive response from the cells during attachment and subsequent proliferation [66]. The favourable and conducive responses of the scaffold to the cells can be further enhanced by modifying properties such as fibre diameter, porosity, and morphology by simply adjusting the electrospinning conditions. Further enhancement of the electrospun material can also be achieved by modifying the fibres to resemble the ECM at the nanometre scale level. This technique involves coating the polymeric fibres with collagen macromolecules, but this process is still evolving, and there are still many challenges ahead.

The phase separation/emulsification technique has been used to produce scaffold structures using a suitable polymeric material [161, 162]. This method is based on the principles of phase separation, for example, PLGA is dissolved in methylene chloride and then by adding water produces an emulsion. This mixture is then poured into a mould and freeze-dried, during which both the water and methylene chloride are removed leaving a highly porous scaffold structure [163]. Recent developments in this technique have produced scaffolds with nanometre scale topographical features abrading the surface, which have resulted in an enhanced cell response to this material [126, 164, 165]. Similar investigations carried out by Ma have revealed that nanopolymers produced using this technique have a distinct advantage in terms of the increased surface area and the resulting enhanced 3D connectivity for various cell types used in tissue engineering applications [88, 166]. Other techniques such as micelle lithography, polymer de-mixing, chemical vapour deposition, chemical etching, and anodization have all been used to produce scaffolds with topographical features capable of soliciting a positive cellular response [35, 167–170]. 

## 4. Cell Response and Behaviour to Surface Topography

### 4.1. Micrometre Scale Topography

With the advent of tissue engineering in the mid-1980s, various fabrication techniques have been used to produce a variety of cell culture substrates and scaffolds to meet the high demands for regenerated tissues. Since then there has been a considerable amount of research into the interactive relationship between cells and micrometre scale surface topography of substrates and scaffold structures. Many studies have reported the influence of submicrometre to nanometre scale topographical features, which are smaller than the size of the cell, on cell adhesion, migration, proliferation, and morphology. These features have size ranges that are similar to the size and topography of proteins found in the ECM [171–179]. And recently, Dalby et al. showed that the interaction of fibroblast cell filopodia with surface features as small as 10 nm directly influenced cell adhesion [180]. Being able to create precise topographical features has made it possible for researchers to investigate the interaction between surface topography and cellular behaviour such as contact guidance [181–187]. The most commonly studied topographical feature is the groove, and its dimensions can provide physical signals to orientate the cell. For example, human gingival cells seeded in micrometre scale grooves fabricated in a silica substrate produced cellular growth that was aligned with the grooved topography [154]. Subsequent investigation found that increasing the depth of the groove enhanced cell alignment, while increasing groove width produced less cell alignment [174]. In addition, in a similar study, mesenchymal cells grown on quartz substrates attach to the ridges of the grooves and then spread via bridging the ridges. The cells not only aligned themselves parallel with the grooves, but they also migrated between 3 to 5 times faster than those on smooth level surfaces [188].

The influence of the topographical feature can have a significant effect on the cell and in some cases dominate other substrate signals such as surface chemistry. Neurons cultured on substrates with a number of protein (laminin) tracks, orientated perpendicular to a range of grooves with varying depths revealed that the neurites aligned themselves with the protein cue when the grooves were shallow. However, when the groove depths were greater than 500 nm the topographical feature became the dominant influence on cell alignment [189]. Furthermore in a similar study, the influential dominance of topography in manipulating and orientating cells was further demonstrated when osteoblasts were aligned by surface grooves rather than chemical cues [190]. Not all cell lines orientate themselves with the groove or ridge, this behaviour is dependent on the topography and the cell type. For example, embryonic rat spinal cord and hippocampus neurons cultured on grooved patterned quartz substrates revealed that neurite spreading was governed by dimensions of the groove and the cell type. The neurites of the spinal neurons tended to orientate themselves with the geometry of the groove, while the neurites of the hippocampus neurons tended to grow perpendicular to shallow grooves and align themselves with deep wide grooves [191]. Similar cell response studies have investigated different surface topographical features such as gratings, posts, fibres, pores, wells and spheres [114, 150, 174, 192]. For example studies by Green et al. showed that a smooth substrate surface covered with evenly distributed 5 *μ*m diameter pores enhanced the proliferation of human fibroblast cells compared to a similar surface with pore diameters of 10 *μ*m [185]. While a study investigating the diameter and depth of a micrometre scaled well pattern evenly distributed on a smooth polydimethylsiloxane (PDMS) substrate found that by increasing the well diameter produced decreasing numbers of fibroblasts attaching to the surface [193]. 

### 4.2. Nanometre Scale Topography

Various studies have shown that nanometre scale topography is an important factor that influences protein adsorption, which in turn mediates the interaction between the cellular environment and the surface of the biomaterial [194–198]. Nanometre scale topography tends to biomimic the cell and modulating signals that are normally found in the 5 to 200 nm features that form part of the ECM structure [114]. The conformation of the adsorbed protein layer on the surface is critical for cell integrins to effectively interact with the underlining surface topography. These interactions are complex and result from local changes in surface properties such as chemistry, protein orientation and adsorption, wettability, and roughness [199]. Effects from surface features such as pits, pores, fibres, grooves, gratings, and spheres all increase the surface area and roughness of the substrate, which influences surface wettability, changes the local surface chemistry, and influences protein adsorption to the nanometre scale surface topography [200]. In addition, proteins with dimensions similar to those of the nanometre scale topographical features are not structurally changed by the surface, while surface features that are smaller or greater than the protein tend to influence the structural arrangement of the proteins during adsorption. This results in the proteins conforming to the nanometre scale topography of the surface [201]. Also, nanometre scale topographical features such as grooves, gratings, pits, and pores have the ability to provide sites for protein deposition, which can enhance contact guidance to cells during attachment [202–205].

**Figure 3:**
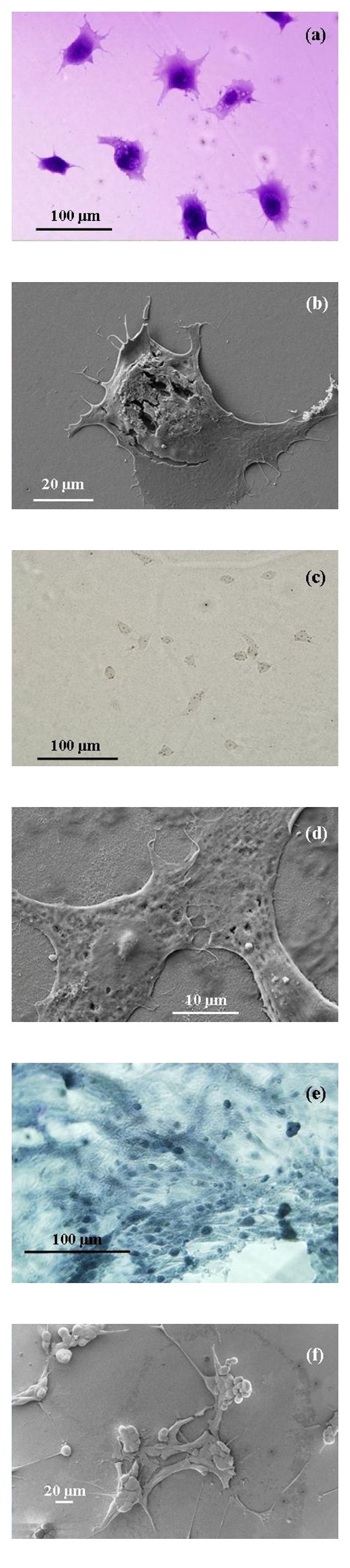
Optical (left) and field emission scanning electron microscopy (right) images of Cos-7 (a, b), Vero (c, d), and Madin-Darby Canine Kidney (e, f) cell lines showing attachment to the electrochemically engineered Anodic Aluminium Oxide membrane. (Images taken by Murdoch Applied Nanotechnology Research Group).

The ability of cells to recognise and interact with nanometre scale surface topography has been examined using a variety of cell types and different surfaces and surface features. For example, Figure 3 presents optical and field emission scanning electron microscopy images of Cos-7, Vero and MDCK cell lines cultured on a self-organized hexagonal array of 110 nm diameter pores, electrochemically formed in a porous anodic aluminium oxide membrane [169]. Endothelial cells cultured on polymer demixed substrates produced a surface covered with islands of nanometre scale heights of 13, 35, and 95 nm [206]. The 13 nm high islands were found to have the most significant effect in assisting cell spreading, while the effect of the 35 and 95 nm high islands was less favourable. The cells found on all three surface modifications exhibited normal cell morphology and responded favourably to the nanometre scale topographical features, with proliferation rates higher than on an equivalent flat control surface [207]. Topographical features on the surface of the substrate can also create physical barriers for cell-to-cell contact or the surface features that could restrain cell spreading and influence cell morphology. The importance of cell-to-cell contact is clearly demonstrated when endothelial cells in contact with neighbouring cells produce significantly higher proliferation rates than single cells without neighbour contacts [208]. Epithelial cells cultured on a silicon oxide substrate covered by 70 nm wide nanogrooves spaced at 400 nm apart, produced by electron beam lithography, were found to align along the ridges of the groove. The cells appear to adhere to the surface topology using the adhesion properties of the lamellipodia and filopodia, which enables the cells to spread over the substrate [204]. Fibroblast cells have also been shown to respond to topographical surface features on a textured substrate's surface. Silica nanoparticles placed onto a substrate to increase the nanoroughness profile of the surface directly influences the behaviour of the cell's adhesion. Studies by Cousins et al. have found that the surface nanoroughness directly influences cell morphology, reduces cell adhesion, and reduces proliferation [209]. In comparison the response and behaviour of fibroblast cells changes with island topographical features. Thus fibroblast cells cultured on polymer demixed substrates with surface covered with islands of nanometre scale heights of 10 and 13 nm have been found to enhance cell adhesion and promote proliferation [210]. However, when 50 and 95 nm high islands were examined the topographical features sustained significantly less fibroblast cell adhesion than the flat control surface used. This clearly demonstrated that the size dependence of the nanometre scale topographical feature influenced the response and behaviour of the fibroblast cell [180]. On the other hand, macrophage cells cultured on a fused silica with 10 to 282 nm nanogrooves induced greater phagocytosis than cells grown on flat control substrates. Macrophages also tended to adhere and spread perpendicular to the direction of the grooves in the substrate [176]. Surface topographical features can also influence the behaviour of osteoblast cells, with many surface features and textures inducing greater cell adhesion and proliferation [211]. In the case of an alumina substrate surface with nanometre scale topographical features, osteoblast cell numbers were up to three times higher than a flat control alumina substrate [20, 212, 213]. In addition, the morphology of these osteoblasts can be modulated by specific nanometre scale topographic features such as grooves, which cause the cells to elongate and align themselves along the axis of the groove [214]. 

### 4.3. Influence of Micrometre and Nanometre Topographical Features on Cells

Surface topography of a substrate can present both micrometre and nanometre scale features to cells that can induce changes within the cell. Studies have shown that cells respond differently to surfaces with ordered topographical features compared to surfaces with a disordered topographical landscape with a similar roughness value. For example, a recent study by Ball et al. revealed that the response of osteoblasts to ordered micrometre scale topography was an increase in metabolic activity compared to osteoblasts on disordered micrometre scale topographical substrates [111]. The results of the study indicate the cellular responses to the substrate surfaces were influenced primarily by the topography of the surfaces, and cells on the ordered surfaces could spread or elongate successfully whilst those on the rough surfaces were constrained. In the case of multipotent mesenchymal stem cells (MSCs) a certain amount of nanometre scale disorder has been found to stimulate MSCs to produce bone minerals *in vitro* in the albescence of any osteogenic supplements [121, 123]. However, there is conflicting work in the literature to whether an ordered topography or a disordered topography is superior in soliciting a favourable cellular response and for inducing effective protein adsorption. 

The conflicting research results in the research probably stem from trying to replicate the ECM of the particular cell type. The tissue engineered scaffold is an attempt to recreate the ECM with all of its complicated signals and stimuli that effectively solicit the appropriate cell response. The surface topographical features whether ordered or disordered are only part of creating a tissue scaffold that completely replicates the nature tissue environment of a specific cell type. And as discussed in this review each cell type responds differently to various topographical features both at the micrometre and nanometre scales. It may be the case that some cell types such as osteoblasts may respond better to an ordered topographical landscape with fixed dimensional features than a disordered or random topography. This may not be the case for all cell lines and it is only with future studies that examine various substrate topographies and materials will the ideal tissue scaffold be found that is cell specific, provides optimum protein adsorption and provides the most effective biochemical and biophysical signals to induce cellular response. 

## 5. Conclusion 

Topographical features on the surface of a suitable substrate can directly influence and affect cellular behaviour. The landscape of the substrate can influence cell adhesion, surface orientation, morphology, cytoskeletal development, differentiation, and proliferation. The surface topography at the nanometre scale tends to induce cell-protein responses which are similar to the signals given by its native ECM. The conformation of an adsorbed protein layer on the surface is critical for cell receptors to effectively interact with the underlining surface topography. Substrate surface topography at the micrometre scale, which has dimensions comparable to cells, has the ability to influence cell adhesion, morphology, and contact guidance. Generally, cells do not penetrate surface features such as microgrooves smaller than 2 *μ*m in width or 500 nm in depth [202]. However, this micrometre scale tendency is cell-dependent and can vary between cell types. On the other hand nanometre scale topography influences cell orientation on the substrate [215, 216]. During cell orientation filopodia provide details of the underlying surface nanometre scale topography. However, the recognition of the nanometre scale topography and how this information is signalled and interpreted by the cell is still an area of active investigation. Nevertheless, it is clear that any future substrate or scaffold structure for a specific tissue engineering application needs to take into account the cell type, selection of the most suitable biomaterial, and also take into account the effects of both micrometre and nanometre surface topography to solicit the most favourable cellular response for a successful clinical outcome.
